# Parents’ Perceptions of Children’s Exposure to Unhealthy Food Marketing: a Narrative Review of the Literature

**DOI:** 10.1007/s13668-021-00390-0

**Published:** 2022-03-12

**Authors:** Christine Driessen, Bridget Kelly, Fiona Sing, Kathryn Backholer

**Affiliations:** 1 Deakin University, Institute for Health Transformation, Global Obesity Centre, Geelong, Australia; 2 Early Start, School of Health & Society, University of Wollongong, Wollongong, Australia; 3 School of Population Health, University of Auckland, Auckland, New Zealand

**Keywords:** Parents, Childhood obesity, Food marketing, Food policy

## Abstract

***Purpose of Review*:**

A key driver of unhealthy diets in children is the marketing of unhealthy foods and beverages. Attempts to regulate children’s exposure to unhealthy food marketing through government-led policies are challenged by commercial interests. Parents shoulder the responsibility of counteracting the effects of omnipresent unhealthy food marketing that children are exposed to within the food environment. In this narrative review we aimed to synthesise the evidence over the last 10 years on parents' perceptions of children’s exposure to unhealthy food marketing and parents support for policies to restrict this marketing.

***Recent Findings*:**

The evidence indicates that unhealthy food marketing leads parents to feel undermined in their ability to provide healthy foods to their children. Despite this concern, parents tend to underestimate the levels of exposure to, and impacts of, unhealthy food marketing to their children, especially in the digital ecosystem.

***Summary*:**

The voices and support of parents represent a significant opportunity to accelerate policy action on food marketing. Increasing awareness among parents and caregivers to the high levels and harmful impacts of children’s exposure to unhealthy food marketing, focusing on their right not to be undermined by such action, may drive support for policy change. Further research is needed to understand parents’ attitudes and perceptions related to their children’s exposure to contemporary unhealthy food marketing, specifically in digital environments, and the perspectives of fathers and parents from low and middle-income countries.

## Introduction

The prevalence of obesity is increasing around the world. Globally 13% of adults and over 124 million children and adolescents (6% of girls, 8% of boys) presented with obesity in 2016 [1]. If left unchecked, projections indicate the global number of children and adolescents affected by obesity will reach 254 million by 2030 [2]. Childhood obesity increases the risk of physical illnesses, including a range of serious non-communicable diseases and has negative impacts on quality of life. Children with obesity are at higher risk of low self-esteem, depression and anxiety, coupled with bullying, poorer school performance and social withdrawal [3, 4]. Obesity treatments have shown to have little effectiveness in reversing obesity and as such, the probability of an adult with obesity returning to a healthy weight is extremely low [5, 6]. Childhood obesity is known to track into adulthood with 80% of adolescents with obesity remaining obese throughout their lifetime [7–9]. The prevention of obesity in childhood is crucial.

The causes of obesity are multifaceted and complex, but experts agree that the food environment is a key determinant of population diets and excess weight gain. The food environment can be defined as the “collective physical, economic, policy and sociocultural surroundings, opportunities and conditions that influence people's food and beverage choices and nutritional status [10].” These elements influence food choice, access, acceptability, quality and safety [11]. The food industry influences our food environments through the ubiquitous marketing of unhealthy (high salt, sugar and fat) and ultra-processed foods and beverages. In 2020, the global food and soft drink industry spent in excess of USD$33 billion on advertising [12]. Compelling evidence demonstrates an association between unhealthy food marketing to children and an increase in brand loyalty, attitudes, preferences and consumption of marketed foods and brands [7, 13–16, 17••, 18, 19]. This normalises unhealthy food consumption [20] and drives increased total energy intake [14, 18, 21•] leading to higher body mass index (BMI) and obesity risk [22, 23].

Unhealthy food marketing encourages young children to frequently request marketed foods from caregivers [15, 24]. Known as ‘Pester Power’, this marketing strategy used by advertisers, including via in-store promotions, packaging, placement and displays [16, 25], undermines parent’s desires and intentions to provide their children with healthy nutritious food. It is also argued that unhealthy food marketing not only undermines children’s human right to health, but also undermines their right to be protected from economic exploitation [26].

Leading public health experts and heath bodies including the World Health Organisation (WHO) recommends governments implement policies to protect children from the harmful impacts of unhealthy food marketing [13, 15, 16, 27–31]. In 2010, Member States of the World Health Assembly unanimously endorsed article WHA63.14 outlining the WHO Set of Recommendations on the Marketing of Food and Beverages to Children [32]. The WHO recommendations stipulated that governments should take an active role to reduce both the exposure and power of unhealthy food marketing. Nevertheless, there has been limited progress on implementing or strengthening government-led policies globally [17••, 33]. Studies of children’s food environments reveal there is limited space where they are not exposed to unhealthy food marketing [17••]. This has led some advocacy groups to describe unhealthy food marketing as “the wallpaper to a child’s life” [34], reaching them via online and offline media, retail environments and places they come together, such as schools and sports clubs [29].

To date, most research on this topic has focussed on children’s potential exposure to unhealthy food marketing on selected media or settings and to a lesser degree, the potential impact of food marketing on children’s short-term food intake. Multiple reviews on these topics provide strong evidence and support calls for policy adoption [13–15, 18]. No recent reviews have synthesised the evidence on how parents experience the issue of children’s exposure to unhealthy food marketing. This is despite parents being the primary mediators between young children’s exposure to unhealthy food marketing and their food intake.

Understanding how parents perceive and experience food marketing can inform advocacy efforts to prioritise government-led actions to protect children from its harmful impacts. In this narrative review, we aimed to synthesise the evidence over the last 10 years on parents' perceptions of children’s exposure to unhealthy food marketing and discuss the implications for food policy advocacy around the world.

Relevant literature was identified from electronic academic databases, which were searched during June 2021. Peer-reviewed journal articles, published in English, since 2010 were reviewed and summarised. This was complemented by a manual search of reference lists of relevant studies and a search of internet search engines to identify grey literature. Reports or peer reviewed studies that included parents’ views, perceptions, attitudes or opinions on children’s exposure to unhealthy food marketing were included.

The studies within this review were mostly from high income countries, typically cross-sectional or repeated cross-sectional study designs or qualitative focus groups. This review narratively synthesises the evidence on parental perspectives about children's exposure to unhealthy food marketing. We explore parents' perspectives regarding specific settings (e.g. sports, retail, schools and outdoor environments), different media (e.g. television and digital media), differences in perceptions among population sub-groups, and support for policy actions to restrict unhealthy food marketing.

## Parental Perspectives About Children’s Exposure to Unhealthy Food Marketing

### Extent of Parental Concern About Children’s Exposure to Unhealthy Food Marketing

Research has indicated that although parents accept their responsibility to guide their children’s food choices [35–39], they may feel undermined or disempowered by unhealthy food marketing [36, 40–44] and support stricter regulatory policies to support them [35, 39, 40, 43, 45–50]. In studies spanning Australia, US, Pakistan, India, UK, Ireland and Indonesia, parents consistently reported feeling concerned about children’s exposure to unhealthy food marketing [36, 39–43, 50–52]. However, the available data and the extent and nature of these perceptions and concerns by parents about food marketing vary across media and settings. 

One of the largest studies examining parental concern for food marketing comes from the US, where an online survey was administered to 2454 parents over 3 years (2009 *n* = 859, 2010 *n* = 797, 2011 *n* = 798) [39]. The authors found parents across a range of sociodemographic groups generally supported policies to reduce unhealthy food marketing to children. Support for policies increased between 2009 and 2011 for regulations to limit unhealthy food marketing to children under the age of 12 on television (60.5% support in 2009 to 66.5% in 2011) and on social media (53.3% to 58.8%). Some of these findings were updated by the same research centre in another annual study over 4 years between 2012 and 2015 with 3608 US parents (2012 *n* = 902, 2013 *n* = 902, 2014 *n* = 906, 2015 *n* = 898) [40], revealing support for restricting unhealthy food marketing on television to under 12 increased further in 2015 to 74.9%. Importantly, 73.5% of parents agreed these restrictions should extend to children under the age of 18. This same study highlighted the widespread concern among parents about the difficulties of raising healthy children in the current food environment [40]. Comparing these earlier and later studies, parents’ perceptions that food and beverage marketing across all categories (all media types, billboards, in-stores, sporting events, toys/giveaways etc.) impact children’s eating habits was higher in 2015 compared to 2009. Concern for unhealthy food advertising was particularly pertinent for online and internet advertising through interactive platforms, such as advergames, where concern changed over time (on a scale of 1 (strongly disagree) to 10 (strongly agree)) from 4.1 (disagree) in 2009 to 6.2 (agree) in 2015). Concern for internet/banner advertisements also increased from 3.8 to 6.0, as did concern for food advertising through social media (4.1 to 5.9) and viral marketing (3.4 to 5.3). Parents feeling pressured from pester power was also reported to increase between 2012 and 2015. Parents’ overall concern for the impacts of food marketing on children was similar to their concerns of how the portrayal of alcohol use in the media may impact children and was moderate compared to other youth-oriented issues in the media. For example, parents were more concerned about the representation of sex, violence and materialism in the media compared to food marketing.

Despite high levels of parental concern about children’s exposure to unhealthy food marketing, and objective evidence demonstrating high exposure to unhealthy food marketing to children across the globe [53••], the studies indicated parents underestimate children’s exposure to unhealthy food marketing and overestimate healthy food marketing exposure [39, 40].

In an Australian study in 2014 [49], 235 Australian parents responded to an open-ended online survey question about their attitudes towards food advertising and children’s diets. Overall, 78% of respondents perceived unhealthy food advertising to have a negative impact on children’s diets. Parents with a high locus of control indicated parents should use more control to counteract the effects of advertising. These parents were typically of normal weight and watched lower levels of television. Parents with a low locus of control reported a desire for policy to restrict advertising. These parents reported higher levels of television viewing and higher body weight (overweight or obesity). Further research, using a larger and representative sample, to understand the different sociodemographic characteristics of those who support and those who do not support food marketing regulations, can inform the targeting of advocacy efforts for civil society mobilisation.

### Third-Person Effect

Studies focussing on food marketing through television and the internet [35, 51] have indicated parents often believe other children are more influenced by unhealthy food marketing than their own children; a concept known as the third-person effect. The third-person effect predicts that personal biases lead to the belief that other people will be more affected by negative media messages than themselves [54]. Studies have found that the third-person effect is particularly salient for negative media messages [54, 55] such as violence, smoking, alcohol and, in this case, unhealthy food advertising. Conversely, the third-person effect also predicts that when it comes to positive media messages that are socially desirable and have potentially desirable outcomes, individuals overestimate the effect on themselves compared to others [55]. During a 2014 UK parents’ study which included one question specific to unhealthy food advertising, the authors indicated a third-person effect in relation to responses [51]. Forty-two parents were interviewed and informed of a statistic that over 40% of children are likely to want unhealthy foods after being exposed to online advertising. All 42 parents agreed this was possible and concerning, yet most expressed that their own children would not be affected by online food advertising and not part of that statistic.

Researchers have indicated there may be a behavioural component to the third-person effect. The research suggests the third-person effect may motivate people to support restricting or censoring potentially harmful media messages due to the belief that others may be more negatively impacted [54, 56, 57], such as the advertising of alcohol and smoking [58], gambling and violence [57], and pornography [59]. A 2012 Korean study examining 318 mothers’ perceptions of television food advertisements [35] revealed that although mothers had negative perceptions about unhealthy food advertising on television, they did not believe food advertising was the main influence on their child’s diet or the most important cause of unhealthy eating habits. In three statements regarding the regulation on television food advertising targeted at children, the authors stated that overall, mothers strongly agreed that stricter regulations should be in place. However, mothers disagreed that their children’s eating habits would be healthier if there was a ban on all television food advertising aimed at children. The authors investigated a third-person effect by asking mothers if children of other parents are more negatively influenced by current television food advertising than their own children. They reported the difference between the rate of agreement (47%) and the rate of disagreement (1%) was statistically significant (*p* < 0.01), supporting a third-person effect.

Further research is needed to understand the third-person effects on parents’ views of unhealthy food marketing and whether there are motivating effects to support regulation. Understanding this effect may be an important tool in tailoring research questions and public health messages to understand and create support for regulations for unhealthy food marketing.

### Food Sponsorship and Marketing in Sports

A regular gathering place for children is at community and elite sports [29]. As such, the food and beverage industry use this as an avenue to market their products to children through sponsorship, advertising and endorsements. A 2017 systematic review by Smith et al. [60] revealed children believe sports celebrity food endorsements “legitimize” eating the promoted foods and are a reliable source of nutrition knowledge, leading to the perception that the foods being promoted are healthy and improve sport performance. A 2013 study of 825 parents by Kelly et al. [47] revealed that most parents supported policies to restrict unhealthy food sponsorship of children’s (76%) and elite sports (71%). Of these parents, most (87%) agreed they would still support restrictions even if it resulted in higher sports fees. More recently, a 2020 Australian study by Gonzalez et al. [61] indicated less parent concern for unhealthy food sponsorship in junior community sports. Among the 279 parents surveyed, 78% agreed alcohol should not be given as prizes, rewards or fundraising, but only 42% agreed fast food rewards should not be given. Given the different questions and sampling used, it is not possible to determine if this difference in policy support reflects changing community sentiments.

### Marketing to Children in Food Retail Environments

Supermarkets have long been used to promote products directly to children as children often accompany parents whilst shopping. The supermarket has been described as one of the most important food environments as it is where most of the household food is purchased [62] and has a major impact on peoples’ food purchases [63]. Numerous studies have shown unhealthy foods are more likely than healthy foods to be price promoted, marketed and in more prominent locations in-store compared to healthy foods [64–68]. These tactics increase impulse buying [69] which occurs frequently in retail environments with studies indicating that over 80% of purchases are unplanned [70, 71]. Whilst these marketing tactics are used to influence all shoppers, some evidence suggests that they are used more often for child-targeted foods compared to other products [72].

Food product packaging is a key marketing tool used to attract consumers. Children are thought to be even more susceptible to product packaging and branding than adults due to the way they respond to visual cues [70, 71]. Marketers know that child-oriented food packaging is a “powerful communicator” [73], yet the foods within them are known to be disproportionally low in nutritional quality [72]. One study found over 50% of foods targeted for consumption by children, include misleading claims about health and nutrition [71]. The use of fun colourful packaging, branded characters and cartoons have been shown to encourage unhealthy food choice, increase taste preferences among children and create brand awareness and character recognition to set children up as future customers [74, 75]. The way unhealthy food is marketed to children in this way aims to represent food as entertainment [74], undermining parents’ attempts to encourage healthy food choices.

Parents have identified in-store food retail marketing tactics as highly effective at influencing their children [37, 38, 40, 76, 77] and a cause of parent–child conflict [71]. Yet, limited research has investigated parents’ opinions on policies to restrict unhealthy food marketing in food retail environments. A 2013 Canadian study [38] conducted in-depth interviews with 60 parents to understand their views related to child-directed food marketing within the supermarket setting. They found that parents were critical about the messages being sent to children, which were viewed as “exploitive” and undermining children’s health. Parents were also concerned about the possible health impacts of food packaging due to the focus on “fun”, which discouraged children from thinking about the product as food, but as entertainment. Parents were questioned as to whether they thought child-targeted supermarket foods should be regulated, and 53% agreed they should. Another 30% of parents were opposed, and the authors cited that parental responsibility and choice was the most common reason for this. The second most common reason for opposing regulations was parents’ belief that the cartoons and fun packaging were acceptable if the foods were healthy, indicating that some of these parents would support regulation if marketing was specifically targeted to unhealthy foods (as opposed to all child-targeted foods) in supermarkets. The remaining parents were unsure (3.3%), indifferent (1.7%) or thought it would be impossible to regulate (1.7%). In a more recent 2019 study, focus groups with 91 Scottish parents/caregivers to understand their perceptions of voluntary in-store policies limiting unhealthy food at checkouts found most participants supported restrictions on unhealthy foods at checkouts and felt more favourably towards the supermarkets that implemented them [78]. The authors suggested that parents would also likely support further restrictions for in-store marketing techniques (shelf placement, child directed food packaging, price offers etc.) as parents spoke about being “bombarded by manipulative marketing.” In another study, 22,264 adults from 5 countries (Australia, Canada, Mexico, UK and US) were surveyed to examine overall public support (not only from parents) for 3 different initiatives related to product placement in food retail stores: (i) checkouts with only healthy products, (ii) fewer end-of-aisle displays containing unhealthy foods or soft drinks and (ii) more shelf space for fresh and healthier foods such as fruits and vegetables. The highest support was found for promoting healthy foods (72%) with restricting unhealthy foods at checkouts receiving the lowest support (48.6%) [79]. The authors noted a high percentage of neutral responses. Further research is required to understand how to increase civil society support for retail policies that support healthy food retail marketing environments.

### Television Food Marketing

A 2019 study comparing rates of unhealthy food advertising on television across 22 countries revealed that children, globally, are exposed to large amounts of television advertisements for unhealthy foods, despite the existence of policies to restrict this advertising in some countries [53••]. The study also found advertising for unhealthy foods was 35% higher during children’s peak viewing times. Although the move to digital marketing has increased dramatically in recent times, television remains one of the most common mediums for food advertising, with 53% of global food industry advertising budgets invested in television in 2020 [12]. Studies have shown that unhealthy food advertising on television impacts children’s food preferences, brand awareness, attitudes, consumption of unhealthy foods and ultimately their health [22, 23, 80].

Numerous studies on parents’ perceptions of the impacts of food marketing on television to children consistently reveal high levels of concern among parents [35, 40–42, 48, 77]. A 2016 study from India interviewed 480 parents (240 rural, 240 urban) about their views on the influence of all types of television advertisements directed at children, finding the most important concern parents had was for “junk food advertisements” [42]. When asked if children usually demand junk food they have seen in television advertisements, parents generally agreed (with an average score of 3.51 among rural parents on a 5-point scale (where 1 = strongly disagree and 5 = strongly agree) and 3.96 among urban parents). Parents (*n* = 167) in a 2011 study from Croatia [48] completed a questionnaire using a 5-point scale (1 = strongly disagree, 5 = strongly agree) on their perceptions of food advertising on television aimed at children. Parents agreed that the food advertised to children contained too much fat and sugar (mean score, 4.10), and advertising of all unhealthy foods should be prohibited (mean score, 3.96). A 2012 Korean study examining 318 mothers’ perceptions of television food advertisements [35] revealed most held negative views of unhealthy food advertising directed at children. Seventy-seven percent of mothers agreed that television advertisements encourage their children to want foods they do not need, and 74% agreed there were too many food advertisements on television directed at children [35].

### Digital and Online Food Marketing

Children are moving away from commercial television viewing [81] and are accessing entertainment via on-demand media, online platforms, social media and gaming, typically via mobile handheld devices. The ways in which children engage with the world today have evolved and moved forward with advancing technologies yet the policies to protect children from harm have lagged allowing the surveillance, harvesting and collection of vast quantities of personal data for targeted online marketing. Global marketing expenditure by the food and beverage industry for online advertising is rapidly increasing, with nearly 25% of the global food advertising spend directed towards internet advertising alone in 2020 compared to only 10% in 2015 [12].

Whilst the evidence is clear that parents are concerned with the unhealthy food advertising that they can see their child viewing (for example, in supermarkets, sporting events and on television), concern or awareness for food advertising on digital devices, which is less visible to parents, appears to be lower than for food marketing through other media and/or settings [36, 40, 43, 49, 50]. Data from the 2015 US study from Harris et al. [40] revealed parents were more concerned about the impact of product placement and movie commercials than they were about food advertising on the internet, websites and on social media. A 2014 UK study involving in-depth interviews with 42 parents about their understanding and opinions of all online advertising, revealed although 64% were concerned about the “profound impact” advertising in general has on children, there was limited parental awareness and concern of online advertising to which children are exposed [51]. The author commented that the lower concern about online advertising (of any products) to children suggests marketers are “getting away with” targeting children without parents knowing [51].

The relatively low parental concern for unhealthy food marketing through digital devices is at odds with the research suggesting that digital food marketing is more pervasive compared to other media and settings, which parents have reported feeling highly concerned about [50]. In a 2016 mixed methods online study with 32 Irish parents [50], 61% believed teens should not be exposed to unhealthy food advertising, yet only 25% of parents were aware that these products were advertised online to children (despite 56% reporting being aware of unhealthy food advertising though television). Prior to being shown examples of online unhealthy food advertisements that children were typically exposed, the authors described parents in the study as being “largely unaware of digital food advertising and effects it may have, and initially were largely indifferent to the issue of digital food marketing.” After parents were shown examples of unhealthy online food advertising, 67% said it had changed their views about online food and drink marketing to children and teens, and 83% agreed the policies banning unhealthy food advertising on television to under 18 should also apply online. A 2018 survey [43] with 140 UK parents reported 87% of parents agreed there were no circumstances where it was acceptable to advertise unhealthy food products to children, yet only 16% reported being concerned about social media and online food advertising. Parents were mostly concerned with unhealthy food advertising on television (56%) and product packaging (49%) compared to food advertising online (16%) and through social media (16%) [43]. In contrast, a small Australian study (2 interviews with 13 parents) in 2014 that focussed specifically on food marketing to which children are exposed through non-broadcast media reported parents were very concerned about online and digital food advertising [36].

As the digital environment continues to evolve at a rapid pace, more contemporary studies are required to gain an in-depth understanding of parents’ attitudes and concerns relating to unhealthy food marketing through digital devices. 

### Schools and Outdoor Advertising

Other common settings where children are exposed to unhealthy food marketing are at school (sponsorships, packaging, canteens, fundraisers), and outdoor public spaces (e.g. using billboards, on public transport, signage) [82]. A 2017 New Zealand study with 168 children using wearable cameras found that children are exposed to approximately 27.3 unhealthy food advertisements every day, with most exposure occurring in the home (33%), in public spaces (30%) or at schools (19%) (the study excluded convenience stores and supermarkets and had limited capture of digital and television media) [83]. In a 2018 UK survey [43], parents (*n* = 140) were asked to indicate the 3 settings (out of a list of 13) of most concern for unhealthy food marketing. Parents rated outdoor settings 5th and promotions or sponsorships in schools 6th (behind television advertising, television characters used on product packaging, toys sold with unhealthy foods, and in-store displays and packaging). Strong support for school-related polices to restrict unhealthy food marketing within US schools was revealed in the 2015 study by Harris et al. [40]. Sixty-four percent supported policies to restrict unhealthy food marketing aligned with school fundraising, 66% supported policies to restrict food marketing more broadly in schools, and 63% supported restrictions for unhealthy food marketing around schools, such as on billboards and buses and reducing the number of fast-food restaurants in close proximity to schools.

### Differences in Parents’ Perceptions Among Population Sub-Groups

Recent research has found that youth from minority groups and lower socioeconomic backgrounds are exposed to and targeted with, more food advertising in the media and their communities [81, 84•]. In the 2015 study by Harris et al. [40] from the US, lower income parents as well as Black and Hispanic parents perceived unhealthy food marketing to impact their children's eating behaviours more than other parents [40, 77]. Those who had a child who was overweight also reported unhealthy food marketing negatively impacted their children’s eating behaviours more than other parents.

Differences in willingness to advocate for stricter food marketing policies have also been observed across population groups. For example in the US, lower income parents were less likely to indicate they would participate in actions to reduce unhealthy food marketing to children (such as signing petitions, sending letters, serving on committees) compared to higher income parents [40]. This may reflect differences in the perception of whether policy change is feasible and/or the perception of whether their voices would be heard. For example, in a study by Tatlow-Golden et al. [50], although parents supported regulating unhealthy food marketing to children and teens through online platforms, a number of parents questioned the practicality of it. However, whether this differs according to higher and lower income parents is unclear. This research highlights the need for more robust empirical evaluations of existing and newly adopted food marketing policies and effective communication of results to the public.

Lastly, a major gap within the literature is the voice and experiences of fathers. With the exception of one 2014 study researching Pakistani fathers’ views of television food advertising aimed at children [41], the majority of respondents included in the available studies were mothers. There is a distinct lack of views and opinions of fathers on children’s exposure to unhealthy food marketing, contrary to their involvement and influence in modern day feeding practices [85].

## Conclusion

Parents consistently report that unhealthy food marketing undermines their attempts to provide healthy and nutritious foods to their children. Parents who are more aware of the extent of food marketing to children and understand its harmful impacts are more likely to support policy action to reduce children’s exposure to unhealthy food marketing [86]. The research to date indicates parents may underestimate the levels of exposure and impacts of unhealthy food marketing to their children, especially in the digital ecosystem. Whilst parents generally support policies to restrict children’s exposure to unhealthy food marketing, there is less concern about this marketing through digital devices.

To adequately protect children from the harmful impacts of unhealthy food marketing a comprehensive government-led legislative response is required. Although some governments have progressed with legislative responses to the issue in recent years, such as Chile [87] and the UK [88], greater policy action is required globally. The voices and support of parents play an integral role in accelerating this action as the lack of demand from civil society for policy change is a key barrier for action on food policy [89]. Parents and other caregivers have the power to hold governments and food companies to account for their actions and inaction [89], and their support for policy has the potential to increase political will. Yet so far, their voices have been largely ignored with limited research available on contemporary parents’ attitudes and perceptions related children’s exposure to unhealthy food marketing, specifically the digital and online environments, and importantly the opinions of fathers. There is also limited research on parents’ opinions from low and middle income countries where unhealthy food marketing is even more ubiquitous, and regulations are often weaker compared to high income countries.

Creating parental support for policy represents a significant opportunity for policy change to reduce the health impacts of unhealthy food marketing to children and support parents in their efforts to raise healthy children. It is essential that the research is communicated effectively and widely to create loud and impactful support for policy action. Increasing awareness among parents and caregivers to the high levels, and harmful impacts, of children’s exposure to unhealthy food marketing, with a focus on their right not to be undermined by such action, may just be the catalyst that wakes civil society [89] into action.
